# Design and Implementation of an Intensive Care Unit Command Center for Medical Data Fusion

**DOI:** 10.3390/s24123929

**Published:** 2024-06-17

**Authors:** Wen-Sheng Feng, Wei-Cheng Chen, Jiun-Yi Lin, How-Yang Tseng, Chieh-Lung Chen, Ching-Yao Chou, Der-Yang Cho, Yi-Bing Lin

**Affiliations:** 1 China Medical University Hospital (CMUH), Taichung 404327, Taiwan; vincentfeng1013@gmail.com (W.-S.F.); 020270@tool.caaumed.org.tw (W.-C.C.); 038767@tool.caaumed.org.tw (J.-Y.L.); 024638@tool.caaumed.org.tw (H.-Y.T.); 022059@tool.caaumed.org.tw (C.-L.C.); 038630@tool.caaumed.org.tw (C.-Y.C.); dycho@tool.caaumed.org.tw (D.-Y.C.); 2 Department of Computer Science, National Yang Ming Chiao Tung University, Hsinchu 30010, Taiwan

**Keywords:** command center, data fusion, Intensive Care Unit (ICU), Internet of Things (IoT), Artificial Intelligence of Things (AIoT), automated machine learning (AutoML)

## Abstract

The rapid advancements in Artificial Intelligence of Things (AIoT) are pivotal for the healthcare sector, especially as the world approaches an aging society which will be reached by 2050. This paper presents an innovative AIoT-enabled data fusion system implemented at the CMUH Respiratory Intensive Care Unit (RICU) to address the high incidence of medical errors in ICUs, which are among the top three causes of mortality in healthcare facilities. ICU patients are particularly vulnerable to medical errors due to the complexity of their conditions and the critical nature of their care. We introduce a four-layer AIoT architecture designed to manage and deliver both real-time and non-real-time medical data within the CMUH-RICU. Our system demonstrates the capability to handle 22 TB of medical data annually with an average delay of 1.72 ms and a bandwidth of 65.66 Mbps. Additionally, we ensure the uninterrupted operation of the CMUH-RICU with a three-node streaming cluster (called Kafka), provided a failed node is repaired within 9 h, assuming a one-year node lifespan. A case study is presented where the AI application of acute respiratory distress syndrome (ARDS), leveraging our AIoT data fusion approach, significantly improved the medical diagnosis rate from 52.2% to 93.3% and reduced mortality from 56.5% to 39.5%. The results underscore the potential of AIoT in enhancing patient outcomes and operational efficiency in the ICU setting.

## 1. Introduction

**Figure 1:**
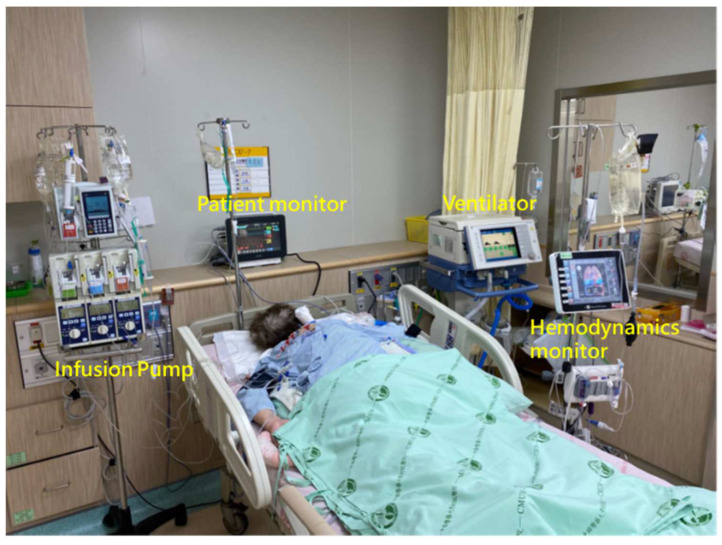
An ICU in the China Medical University Hospital (CMUH).

As the world approaches an aging society which will be reached by 2050 [1], Artificial Intelligence of Things (AIoT) integration is essential for enhancing medical technologies. Currently, over 62% of medical devices are wearable or implantable [2,3], aimed at home or sub-health monitoring. The remaining 38% are primarily used in Intensive Care Units (ICUs) [2,4], an area with less focus on AI application. In hospitals, a significant portion of critical medical devices, including patient monitors, ventilators, infusion pumps, and hemodynamics monitors, are deployed in ICU settings. This is demonstrated in Figure 1, which shows an ICU example. This reflects the need for advanced AIoT solutions in ICUs to aid healthcare providers in managing the increasing demands of patient care in these critical environments.

**Figure 2:**
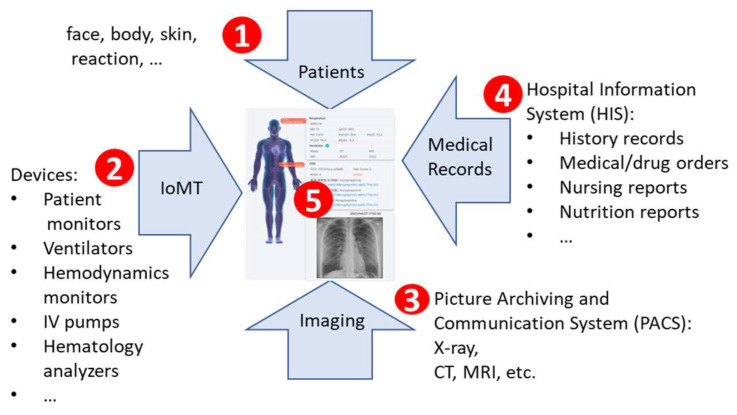
ICU physicians need to integrate a significant amount of medical information.

ICUs are high-stress environments where healthcare professionals are responsible for the continuous monitoring and treatment of critically ill patients. The setting is replete with sophisticated medical equipment and life-support systems. Physicians are inundated with substantial quantities of data, including patient observations (Figure 2(1)), both real-time and historical information from medical devices (Figure 2(2)), laboratory test results, imaging findings (Figure 2(3)), and comprehensive medical records (Figure 2(4)). The complexity of patients’ health conditions, the urgency of required medical interventions, and the variability of the workload contribute to a higher risk of errors in the ICU compared to other hospital departments [5].

Additionally, medical care in the ICU is a collaborative effort involving multidisciplinary healthcare professionals, such as intensivists, cardiac surgeons, respiratory therapists, nurses, pharmacists, and nutritionists. Effective communication between these clinical team members is essential [6]. A transparent, integrated, and comprehensive information center will facilitate such communications, enhancing the overall quality of care.

It is essential to utilize AIoT-enabled Command Center technologies to reduce the workload of critical care staff, improve communication between clinical teams, and enhance patient care quality. The integration of medical AIoT technologies is pivotal for data fusion in the ICU command center, despite the high entry barrier for implementing AIoT in such a complex environment. While interest in AI research related to critical care is growing, comprehensive bibliometric studies assessing scientific output globally are still lacking. Although some insights are available from Tang et al. [7], there is a gap in the literature on the practical application of AIoT in the ICU. This paper aims to bridge this gap by detailing CMUH’s experience in developing an AIoT-enabled command center, using the Respiratory ICU (RICU) as an example to showcase the construction of an intelligent ICU based on this real-world implementation.

## 2. Challenges of AIoT-Enabled ICU

Adapting information fusion and decision-making technologies in the ICU is essential, yet it faces unique challenges. Firstly, strict ICU management often restricts access, with rooms open for limited periods, hindering data collection from devices. Secondly, the complexity of ICU equipment, due to patients’ severe conditions, poses a high knowledge threshold that surpasses that of standard wearable devices. Furthermore, effective medical decision making in the ICU demands the integration of comprehensive data from multiple devices, moving beyond reliance on single-device readings. These factors necessitate a sophisticated approach to data fusion and medical decision making in the ICU setting.

To achieve effective medical decisions, multiple data sources should be fused, a process known as multi-modality AI. These data sources include:

- Patient monitors tracking vital signs such as heart rate (HR), blood pressure (BP), respiratory rate (RR), and blood oxygen saturation (SpO_2_);
- Ventilators managing breathing parameters like tidal volume (Vt), exhalation minute volume (Ve), and inspired fraction of oxygen (FiO_2_);
- Hemodynamic monitors observing circulatory system metrics, including cardiac output (CO) and stroke volume (SV);
- Infusion pumps regulating and documenting medication flow;
- Hematology and blood gas analyzers assessing blood components and gases such as partial pressure of oxygen (PO_2_) and partial pressure of carbon dioxide (PCO_2_).

These integrated data are vital for real-time patient monitoring and treatment adjustment in the RICU.

Apart from combining the medical expertise of physicians with AI model researchers, it is also necessary to integrate the maintenance unit for medical devices (medical engineering department), the medical process improvement unit (medical supply room), the database and system integration unit (information department), and other relevant departments. Therefore, strong support from the hospital is essential.

In the RICU, where patients often battle severe pneumonia and acute respiratory distress syndrome (ARDS), physicians must combine clinical insights with real-time data from medical devices. The advanced monitoring of ARDS patients involves dynamic adjustments of ventilation strategies, informed by calculated parameters like the *ventilatory ratio* [8], *P/F ratio* [9], and low tidal volume ventilation (*LTVV*) [10] based on Equations (1)–(3), which are listed below. These parameters are critical in guiding the treatment and management of ARDS in the ICU.

(1) $$ventilatory\;ratio=\frac{Ve\times {PCO}_{2}\times Vt}{100\times 37.5\times PBW}$$

(2) $$P/F\;ratio={PO}_{2}/{FiO}_{2}$$

(3) LTVV=Vt/PBW

To enhance RICU patient outcomes, particularly for those with ARDS, the calculation of key ventilation parameters using real-time data is essential. The *ventilatory ratio* (Equation (1)) utilizes ventilator outputs (Ve and Vt), PCO_2_ from blood gas analysis, and the patient’s predicted body weight (PBW) [11] from medical records. The *P/F ratio* (Equation (2)) is derived from the PO_2_ measured by blood gas analysis and FiO_2_ from the ventilator settings. *LTVV* (Equation (3)) is calculated using the Vt and PBW.

Research from CMUH RICU physicians over two years has shown that applying these equations can significantly decrease mortality rates in ARDS patients [12]. This success has bolstered the confidence of ICU physicians in AIoT-based command center technologies to reduce the workload of critical care staff, improve communication between clinical teams, and enhance patient care quality.

## 3. Requirements and Specification of CMUH-RICU Command Center

RICU healthcare providers seek a sophisticated command center that reduces their workload by using AIoT technology for diagnostic and predictive analytics. Capable of being operated both on-site and remotely, it offers early alerts for quick intervention in patient care. The COVID-19 pandemic underscored the necessity for ICU command centers, consolidating vital patient data and video for remote medical teams to analyze and act upon [13,14,15,16,17]. The rapid fluctuations in the condition of critically ill patients necessitate a unified platform that merges diverse health data streams, enhancing understanding and patient care quality.

The CMUH-RICU command center, leveraging AIoT, combines real-time and historical medical data with medical records, featuring AI-driven early detection for conditions like sepsis, ST elevation myocardial infarction (STEMI), and ARDS. It boosts outcomes by enabling preemptive measures. Key functionalities of the CMUH-RICU include business intelligence for monitoring ICU and medical device usage, as well as AI services that evaluate critical treatment factors and predict deterioration in physiological indices. These are accessible through a web-based interface on multiple digital devices, providing flexibility and immediate access for medical personnel.

**Figure 3:**
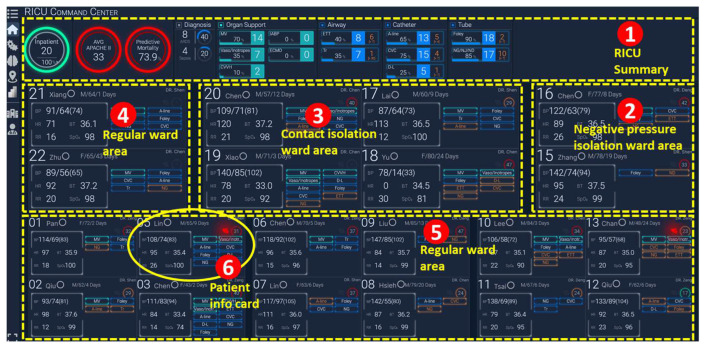
The main panel of the CMUH-RICU command center.

Figure 3 illustrates the main panel of the CMUH-RICU command center, which is divided into five areas. The RICU summary area (Figure 3(1)) provides the unit statistics; including bed capacity (occupied/available); list of physicians (with filtering capabilities); ARDS (number, ratio); Acute Physiology and Chronic Health Evaluation II (APACHE II) (unit average); and medical devices (quantity/usage rate) such as ventilators, hemodynamics monitors, infusion pumps, and extracorporeal membrane oxygenation (ECMO).

The negative pressure isolation ward area (Figure 3(2)) provides patient information on respiratory isolation patients. The contact isolation ward area (Figure 3(3)) provides patient information on contact isolation patients. The rest of the patients are in the regular ward area (Figure 3(4,5)). The patient information includes the vital signs such as HR, BP, RR, SpO_2_ (where the text color turns red if values exceed the normal range), organ support devices (including ventilators, infusion pumps, ECMO), isolation status (antibiotic-resistant bacteria), and alarm notifications (such as extreme laboratory values and AI inference results).

**Figure 4:**
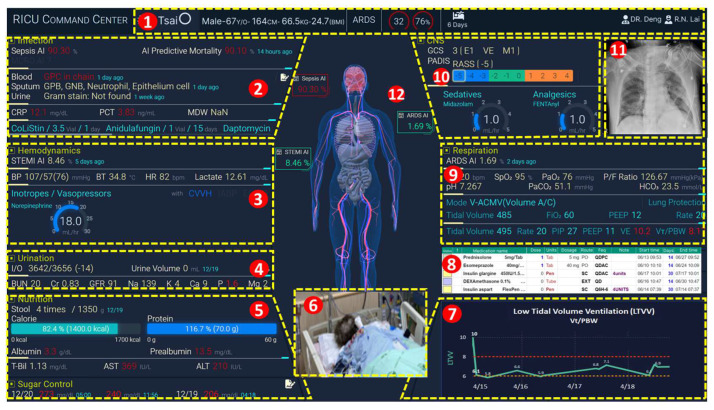
Patient’s digital twin information card.

Personalized care can be accessed through the main panel of the command center, leading to the patient’s digital twin information card. These patient-centric data display the subsystem and important medical status of the patient. In the main panel, clicking on a specific bed (Figure 3(6)) navigates to the patient’s digital twin information card screen. Figure 4 illustrates this screen, with the features described below.

The patient’s basic information (Figure 4(1)) includes their bed number, name, gender, age, principal diagnosis, APACHE II, death rate, ICU length of stay, isolation status, doctor, and nurse. The real-time video (Figure 4(6)) supports real-time monitoring of the patient. The current medication information (Figure 4(8)) includes the medication name, dose, unit, route, start time, and end time. The *LTVV* (Figure 4(7)) can, for example, utilize the Vt/PBW trend to compute Equation (3).

The AI interpretation results (Figure 4(12)) display the current AI inference results, including ARDS prediction, sepsis prediction, STEMI detection, etc., to be elaborated upon in Section 4.3. The screen also includes the key parameters of the hemodynamic monitor (Figure 4(3)), the key parameters of the urinary system (Figure 4(4)), and the nutritional status (Figure 4(5)). The screen also includes a body map (Figure 4(12)) that displays alarms and extreme abnormal test results regarding patient’s organs. The screen also includes the key parameters of the ventilator (Figure 4(9)), such as Vt, Ve, FiO_2_, and PBW, which are used to compute Equations (1)~(3). The screen also includes the infusion pump status (Figure 4(10)). The Picture Archiving and Communication System (PACS; Figure 4(11)) displays the most recent medical images.

Through Figure 3 and Figure 4, multidisciplinary healthcare professionals can easily retrieve related information, shorten communication time, and expedite diagnostics.

Following the therapeutic guidelines for ARDS, it is crucial to improve the patient’s hypoxemia while protecting their lungs. According to a study [18], only 60% of ARDS cases are promptly diagnosed, and only 66% of patients receive lung-protective ventilation. Through the patient’s information card in the CMUH-RICU command center (Figure 4), physicians can access integrated data promptly, facilitating immediate changes in patient management. By harnessing and integrating diverse information from the CMUH-RICU command center, we can achieve optimal therapeutic outcomes.

## 4. Design and Implementation of the CMUH-RICU Command Center

**Figure 5:**
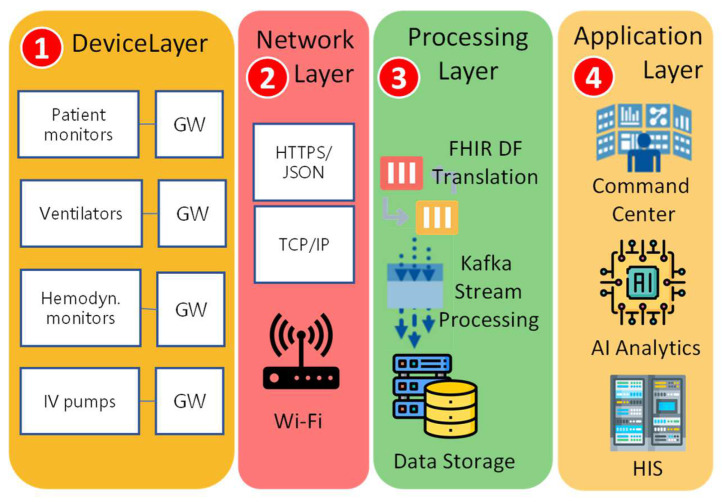
The architecture of the CMUH-RICU system.

The medical crew at CMUH-RICU obtains information through a main panel interfaced by the command center, which is a critical part of CMUH-RICU. The medical data paths supporting user access requirements are structured into four layers as per the reference model [19,20], as depicted in Figure 5:

- Device Layer (Figure 5(1)): comprises medical devices and gateways that facilitate data transmission using interfaces like RS-232, Bluetooth, and Wi-Fi;
- Communication Layer (Figure 5(2)): utilizes the hospital’s Wi-Fi network to connect the gateways to the internal network;
- Processing Layer (Figure 5(3)): handles medical data processing and storage, including format conversion, data stream processing, and managing large data volumes;
- Application Layer (Figure 5(4)): analyzes medical data for user and application access, serving the command center, medical AI, and Hospital Information System (HIS) in the hospital environment.

Under the architecture shown in Figure 5, we establish a data flow from the source end (the device layer) to the sink end (the application layer) to accomplish an intelligent CMUH-RICU.

### 4.1. Protocol Translation in the Processing Layer

**Figure 6:**
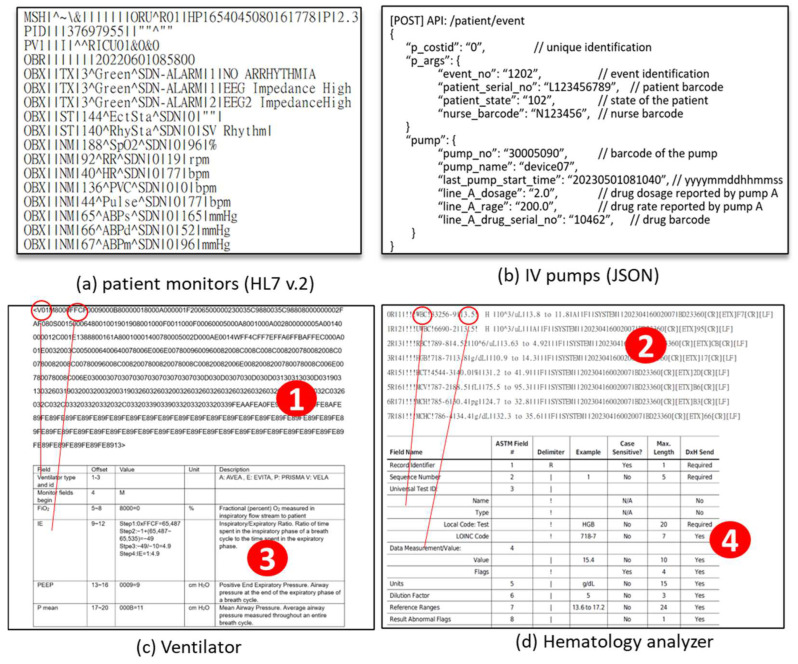
Medical data formats.

**Figure 7:**
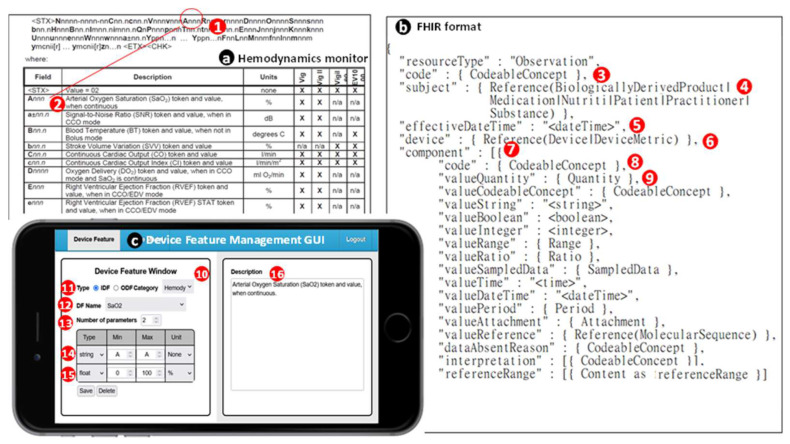
FHIR observation resource in JSON format and its translation for hemodynamics monitor.

In the CMUH-RICU, medical devices use various data formats, including standard formats like Health Level Seven International (HL7) v2 [21] for patient monitors (Figure 6a) and JavaScript Object Notation (JSON) for infusion pumps (Figure 6b), as well as proprietary formats for ventilators (Figure 6c), hematology analyzers (Figure 6d), and hemodynamics monitors (Figure 7a). These proprietary formats, consisting of custom codes and values (e.g., Figure 6(1,2) and Figure 7(1)), require decoding based on the manufacturers’ technical documentation (e.g., Figure 6(3,4) and Figure 7(2)).

Interoperability in healthcare necessitates a unified standard for data exchange across medical applications. HL7, an international standards organization, addresses this need by developing standards like Fast Healthcare Interoperability Resources (FHIR) to facilitate electronic data sharing between disparate healthcare systems, continuing its 20-year legacy of advancing healthcare data and modeling standards [22].

The FHIR standard utilizes the observation data format for exchanging physiological signals, as shown in Figure 7b. This format includes comprehensive field items from which necessary fields can be chosen. The observation’s code field (Figure 7(3)) specifies the observation type, such as vital signs or lab data, and uses Logical Observation Identifiers Names and Codes (LOINC) [23] as the recommended coding system. The subject field (Figure 7(4)) contains the patient identifier, linking to their medical record. The effectiveDateTime field (Figure 7(5)) records the date and time of the observation. The device field (Figure 7(6)) details the device specifics, including type and location. The components field (Figure 7(7)) arrays different physiological parameters like HR and blood pressure.

LOINC codes are advised for coding physiological parameters in FHIR, with measurement units expressed in Unified Code for Units of Measure (UCUM) [24]. For instance, HR has the LOINC code “8867-4” and is measured in “{beats}/min”. FHIR accommodates various value types such as String, Boolean, Integer, Range, Sample data, etc., providing definitions for each.

FHIR utilizes a Representational State Transfer (RESTful) Application Programming Interface (API), enabling easy deployment and supporting information exchange in various formats like JSON and Extensible Markup Language (XML). Taiwan’s Ministry of Health and Welfare endorses HL7 FHIR for its streamlined exchange process, leading CMUH-RICU to adopt FHIR as the standard data format. Medical device data are converted to FHIR via a translation mechanism in the processing layer (Figure 5(3)), using a web-based Graphical User Interface (GUI) for device feature management (DFM; Figure 7c) [25].

DFM maps data formats to FHIR, treating each datum as a device feature (DF). For common formats such as HL7 v2 and JSON, DFM employs GPT-4 [26] to generate mappings executed during data transmission (Figure 5(3)). Proprietary formats provided by vendors in tabular form (Figure 6(3,4) and Figure 7(2)) are converted into DFs using the DFM GUI.

Taking the hemodynamics monitor as an example (Figure 7a), DFs are created from the device’s data specification table. Categories like “Hemodynamics” (Figure 7(10)) are inputted, with specific entries, such as arterial oxyhemoglobin saturation (SaO_2_), labeled as DF names (Figure 7(12)). DFs contain parameters like delimiters and value ranges (Figure 7(13–15)). DFM generates parsing rules from these DFs, which GPT-4 uses with Lex [27] to create the lexical analyzer. FHIR codes for methods and measurements are derived from DF names and parameters, as shown with SaO_2_, coded “2708-6” and accompanied by its data type and unit (Figure 7(8,9)).

### 4.2. Data Flow in the Processing Layer

Medical data flow from the device layer to the network layer and are processed in the streaming platform Kafka, selected for its performance in data pipelines and scalability [28]. MongoDB, a NoSQL database well-suited for JSON (FHIR) data, serves as the database due to its ranking as the top NoSQL database as of April 2024 [29].

**Figure 8:**
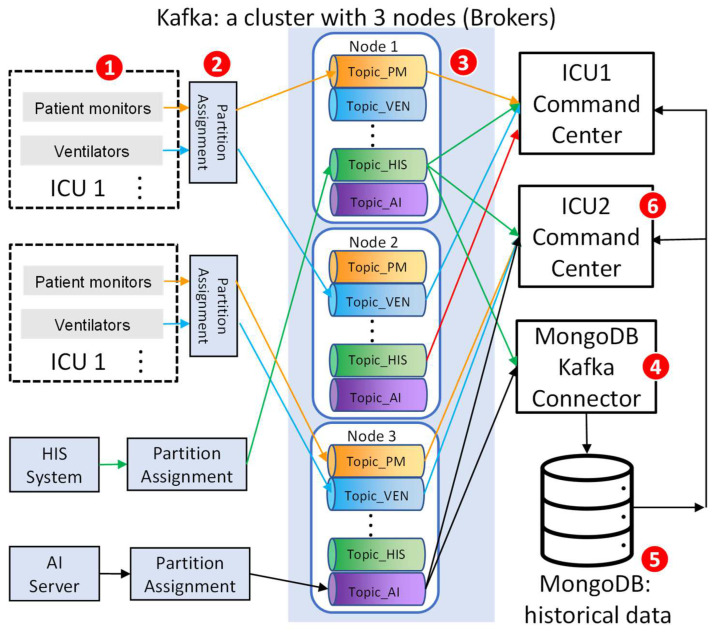
The architecture and data flow of CMUH-RICU in the network and the processing layers.

The CMUH-RICU system architecture, shown in Figure 8, encompasses the device layer (Figure 8(1)), processing layer (Figure 8(2–4,5)), and application layer (Figure 8(6)).

In Kafka, each medical device category corresponds to a “topic”, facilitating the organization and management of data streams. For example, patient monitors use “Topic_PM”, while ventilators use “Topic_VEN”. Kafka servers can operate as a single node or in a cluster, with each node managing distinct message queues for different topics. Messages from the same topic but different devices may go to different nodes’ queues, ensuring load balancing and fault tolerance. The partition assignment strategy (Figure 8(2)) further enhances load balancing and fault tolerance, allowing data rerouting if a node fails.

MongoDB stores historical medical data (Figure 8(5)), which is accessible to the ICU command centers for analysis. The MongoDB Kafka Connector (Figure 8(4)) facilitates the transfer of data from Kafka to MongoDB, with each medical device category’s data stored in a MongoDB collection. Historical data are retrieved by filtering based on patient ID and location, similar to real-time data access.

The designed architecture offers flexibility and scalability, allowing for straightforward integration or removal of data sources without disrupting the existing system. As the quantity of medical devices fluctuates, the impact on queues and ICU command centers is negligible. Changes in device types only necessitate modifications to the corresponding Kafka topics.

Beyond medical data, the CMUH-RICU command center also incorporates medical records from the HIS and images from the PACS, integrating these sources seamlessly using the same framework. The HIS sends individual medical records to the “Topic_HIS” queue, which are merged into a single document within MongoDB for efficient retrieval and utilization at the command center.

### 4.3. AI Incorporation in the CMUH-RICU Command Center

**Figure 9:**
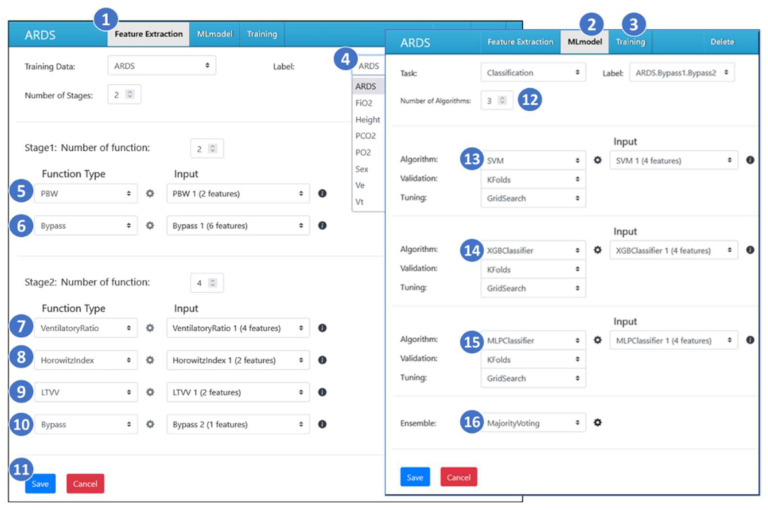
AItalk NCLC approach of the ARDS AI.

CMUH-RICU leverages a no-code low-code (NCLC) machine learning platform known as AItalk [30] (Figure 9) for the development of its AI applications. This platform allows medical experts to define feature extraction processes and machine learning models via a web-based GUI. As depicted in Figure 9, the ARDS AI application interface includes three buttons on a blue banner for setting up feature extraction (Figure 9(1)), machine learning models (MLmodel; Figure 9(2)), and training (Figure 9(3)).

Feature extraction, a critical data pre-processing step in traditional statistics and machine learning, is structured in multiple stages within AItalk. The initial input (Stage 0) consists of “sensor features” from medical devices stored in MongoDB (Figure 8(5)). At this stage, the features such as height and sex are utilized to calculate PBW at Stage 1 (Figure 9(5)), with AItalk containing pre-built functions for PBW based on these inputs. Additionally, six sensor features, including FiO_2_, PCO_2_, PO_2_, Ve, Vt, and the ARDS label, are passed directly to Stage 2 using a Bypass function (Figure 9(6)).

At Stage 2, a combination of features from Stage 1, like PBW, PCO_2_, Ve, and Vt, are employed to calculate the *ventilatory ratio* (Figure 9(7)) using Equation (1). Medical experts can select or input a low-code Python program through a pop-up programming window by clicking on the “Function Type” drop-down list to implement the required calculations.


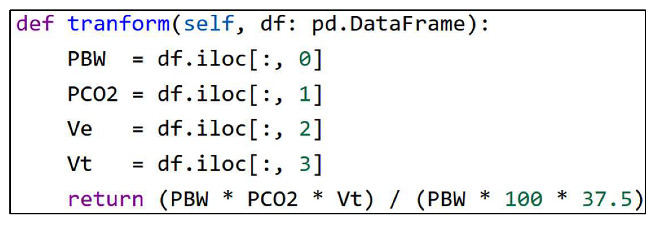

A low-code Python program

Upon completing the feature extraction process, the medical expert can proceed to set up machine learning models for training by clicking the MLmodel button (Figure 9(2)). The task of predicting ARDS is treated as a classification challenge. Experts can choose from a range of algorithms suitable for this purpose (Figure 9(12)), and in the given example, three algorithms are selected: Support Vector Machine (SVM; Figure 9(13)), eXtreme Gradient Boosting (XGB; Figure 9(14)), and Multiple-Layer Perceptron (MLP; Figure 9(15)). Initiating the training with the training button (Figure 9(3)), AItalk runs these algorithms, presenting outputs and metadata for each. The most effective algorithm in this case is XGB, with an Area Under the Curve (AUC) of 0.88, demonstrating strong predictive accuracy for identifying ARDS cases.

These AI services are integrated using the Kafka streaming platform and MongoDB database (Figure 8). The inference results from AI services are pushed to the “Topic_AI” queue and distributed to the CMUH-RICU command centers that need the information.

Additional AI applications in the CMUH-RICU command center include sepsis prediction and STEMI detection. The sepsis prediction application assesses the risk of antibiotic resistance and sepsis onset, analyzing the patient medical history, antibiotic usage, and data from hematology analyzers (white blood cell differential count, nucleated red blood cell, reticulocyte count, etc.). It uses an XGB model with an AUC of 0.861 [31]. For STEMI detection, electrocardiogram (EKG) data are analyzed to identify the likelihood of a STEMI heart attack, employing a convolutional neural network (CNN)–long short-term memory (LSTM) model with an impressive AUC of 0.997 [32].

## 5. Discussion: An ARDS Use Case

To illustrate the effectiveness of information fusion in the CMUH-RICU command center, we will delve into data-level fusion, feature-level fusion, and algorithm-level fusion, focusing on the use case of ARDS.

Data-level fusion integrates medical data, transmitted using the FHIR format, with HIS patient history data, linked by visit IDs to records such as in-patient and drug order records. At CMUH, with 1000 in-patients, this fusion process converts approximately 1.35 million visit records into a patient-centered database in MongoDB. For example, in the calculation of PBW (Figure 9(5)), integration with basic patient information from the HIS is required in order to obtain height information. Database queries first limit the scope to in-patients to obtain visit IDs. These visit IDs are then used to query height information in in-patient records, selecting the latest record. Subsequently, these data are merged with respiratory data from the CMUH-RICU platform to form the dataset used for training the ARDS AI.

Feature-level fusion involves features generated from raw data, as depicted in Figure 9(5–10), serving as inputs for machine learning algorithms. These features hold clinical significance and are less prone to arbitrary automatic generation. In ICU applications, multiple raw patient data are processed and computed into clinically meaningful features, enabling healthcare professionals to make clinical decisions. Hence, the interface in Figure 9(1) is designed for the easy addition of features.

Algorithm level fusion incorporates six features (Figure 9(5–10)) into the machine learning algorithms. The algorithms autonomously learn how to fuse these features within the model. For instance, in SVM, the vector formed by these features undergoes a transformation into a high-dimensional space, followed by classification. Alternatively, in MLP, the vector incorporating the four features undergoes processing through a neural network, involving subsequent forward and backward operations. Additionally, Figure 9(13–15) illustrate the significance of configuring the ratio between the training set and validation set, along with tuning hyperparameters, to achieve optimal fusion and algorithmic performance.

Furthermore, our system includes automatic machine learning (AutoML) functionality. In Figure 9(2), we configure three algorithms: SVM (Figure 9(13)), XGB (Figure 9(14)), and MLP (Figure 9(15)), and employ ensemble algorithms such as voting, averaging, boosting, bagging, stacking, and blending for algorithm fusion. Performance indices can include the F1 score, precision rate, accuracy, etc. The system derives the best model based on the selected performance index. In Figure 9, we adopt majority voting (Figure 9(16)).

The command center and ARDS use cases already utilize these information fusion mechanisms. Let us consider the case of a patient with a history of atrial fibrillation. He developed severe pneumonia, leading to ARDS and septic shock, which required immediate endotracheal intubation. Consequently, he was admitted to the RICU.

**Figure 10:**
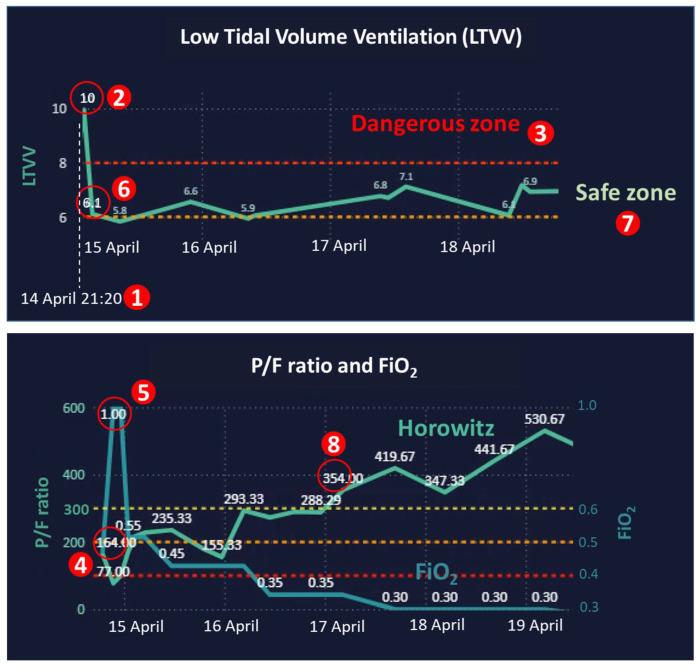
The *LTVV*, *P/F ratio*, and FiO_2_ curves of an ARDS patient.

On 14 April at 21:20 (Figure 10(1)), the patient was diagnosed with ARDS with the assistance of ARDS AI prediction; his *LTVV* was 10 (Figure 10(2)), which is higher than 8, the maximum safe *LTVV* value (Figure 10(3)). The *P/F ratio* was 164 (Figure 10(4)), which is lower than 300, the minimum safe value. Additionally, the FiO_2_ was 1.0 (Figure 10(5)), exceeding the safe value, which must be lower than 0.4. Upon receipt of the alert from the command center, the critical care team, composed of intensivists, cardiac surgeons, respiratory therapists, nurses, pharmacists, and nutritionists, worked together to provide care. Coupled with an effective treatment regimen of specific antibiotics and antivirals, the patient’s condition showed significant improvement within 6 h. On 15 April at 03:30, his *LTVV* value decreased to 6.1 (Figure 10(6)), and it continued to remain within the safe zone (Figure 10(7)). Additionally, his *P/F ratio* and FiO_2_ showed improvement, both falling within the safe range on 17 April (Figure 10(8)). Following a two-week hospitalization, he made a complete recovery and was discharged successfully.

The integration of data-level, feature-level, and algorithm-level information fusion within the command center has significantly enhanced ARDS diagnosis and management, reducing the burden on clinicians and improving patient outcomes. Compared with traditional care practices, this approach increased the ARDS diagnosis rate from 52.2% to 93.3% and decreased mortality from 56.5% to 39.5%, demonstrating a potential life-saving impact for one in every five ARDS patients.

## 6. Discussion: Delay Time and Fault Tolerance Performance

In the CMUH-RICU, numerous medical IoT devices transmit a large volume of data streams. Efficiently aggregating data from different sources, ensuring data integrity, and providing it to various applications are crucial tasks. This section examines the performance of data bandwidth and the fault tolerance design of CMUH-RICU.

### 6.1. Throughput and Delay Analysis

In CMUH, the estimated data flow and storage capacity of existing medical IoT devices in all ICUs are:

- Patient monitors: approximately 470 units with a data flow rate of 29.7 Mbps, requiring a storage capacity of 8.7 TB per year;
- Ventilators: approximately 180 units with a data flow rate of 26.1 Mbps, requiring a storage capacity of 11.3 TB per year;
- Hemodynamic monitors: approximately 20 units with a data flow rate of 2.32 Mbps, requiring a storage capacity of 630 GB per year;
- Infusion pumps: approximately 500 units with a data flow rate of 344 Kbps, requiring a storage capacity of 1.1 TB per year;
- Hematology analyzers: approximately 30 units with a data flow rate of 7.2 Mbps Kbps, requiring a storage capacity of 6.8 GB per year.

**Figure 11:**
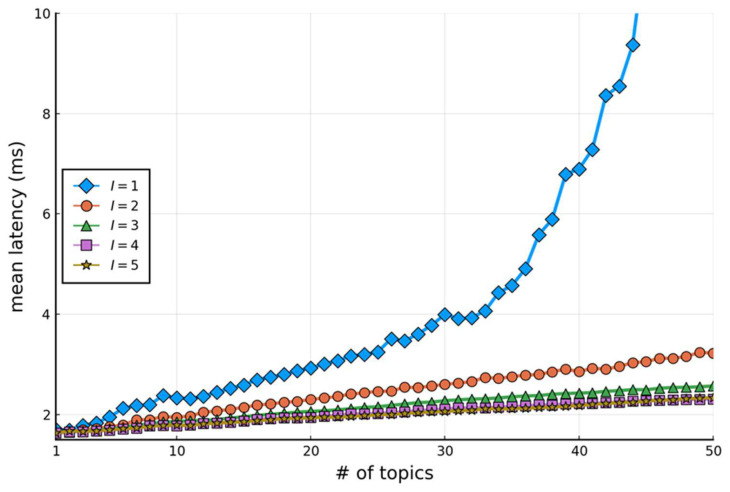
The expected delay time of the Kafka data path.

The net data flow rate of these five topics is 65.66 Mbps, requiring a net storage capacity of 22 TB per year. Based on the above workload, Figure 11 plots the expected delay time of the Kafka data path when the cluster has n nodes (1≤n≤5) and the number of topics increases from 1 to 50. The figure demonstrates the advantage of load balancing, where the delay decreases as n increases. In the current implementation, we select n=3 to process five topics with an expected delay of 1.72 ms. It can also handle up to 50 topics with an expected delay of 2.57 ms.

### 6.2. Fault Tolerance Analysis

In terms of fault tolerance analysis for the Kafka server, we need to verify the condition wherein the server is fault-tolerant when the number *I* of nodes in the Kafka server is three. Specifically, we compare the performance results for *I* = 2 and *I* = 3.

**Figure 12:**
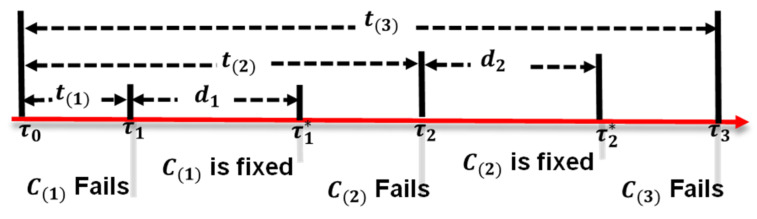
The timing diagram of the Kafka server cluster with *I* nodes.

Consider the Kafka server cluster with I nodes. Suppose that Node $C_{i}$ operates normally for a period $t_{i}$ and then fails. Let $t_{\left(i\right)}$ be the order statistics of $t_{i}$, where ${t_{\left(1\right)}\leq \ldots \leq t}_{\left(i\right)}\leq \ldots \leq t_{\left(I\right)}$ (1≤i≤I). Figure 12 is the timing diagram for *I* = 3. In this figure, Node $C_{\left(i\right)}$ fails at time $\tau_{i}$ and is fixed at $\tau_{i}^{*}$, where $t_{\left(i\right)}=\tau_{i}-\tau_{0}$ and $d_{i}=\tau_{i}^{*}-\tau_{0}$. If we can repair Node $C_{\left(i\right)}$ within the period $d_{i}$, where $t_{\left(i\right)}+d_{i}\leq t_{\left(I\right)}$, then we guarantee that the system can continue to operate normally and is fault-tolerant. Let $P_{F}(I)$ be the probability that the I-node Kafka server cluster fails. For I=2, the system fails with the probability $P_{F}\left(2\right)=1-\mathrm{Pr}\left[\tau_{1}^{*}{\leq \tau }_{2}\right]$. Assume that both $t_{i}$ and $\tau_{2}$ have exponential density functions. Then, only the expected value, $E\left[t_{i}\right]=\frac{1}{\lambda },$ and $E\left[\tau_{2}\right]=\frac{1}{\beta }$ have an impact on $P_{F}(I)$. This is called the mean value analysis on $t_{i}$, which allows us to quickly estimate the performance of the system through the means of its parameters. Let $\gamma =\frac{\beta }{\lambda }$; then, from the tedious derivation in [33], we obtain very simple forms for $P_{F}\left(I\right)$, where

(4) $$P_{F}\left(2\right)=\frac{\gamma }{\;1+\gamma }\;\;and\;\;P_{F}\left(3\right)=\frac{2\gamma^{2}}{\left(2\gamma +1\right)\left(\gamma +2\right)}\;\;\;$$

**Figure 13:**
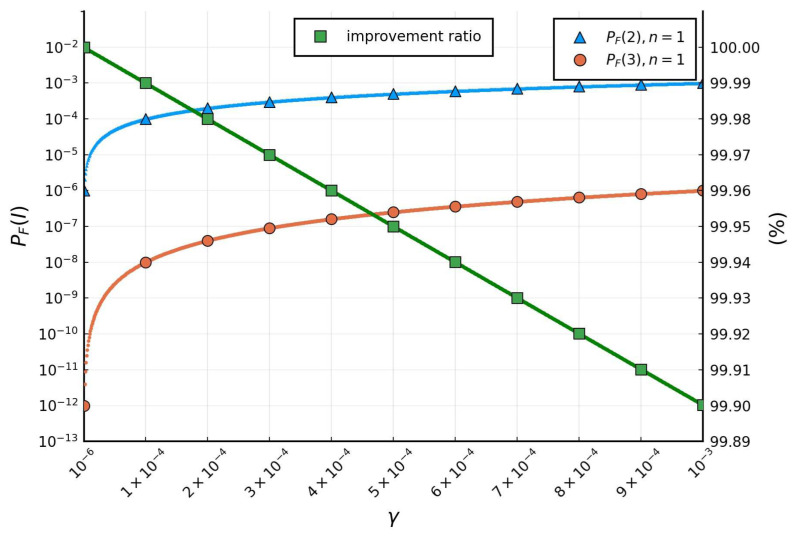
Effect of *I* on $P_{F}$*(I)*.

Based on the above derivations, we plot Figure 13 to ensure prompt resolution within 9 h for a one-year node lifespan.

The figure indicates that, as the number of nodes increases from 2 to 3 in a cluster, $P_{F}\left(I\right)$ drops significantly, i.e., the improvement is $\frac{P_{F}\left(2\right)-P_{F}\left(3\right)}{P_{F}\left(2\right)}\geq 99.9\%$. Also, if the expected life $E[t_{i}]$ of a node of the server is about one year, then the sever almost never fails (i.e., $P_{F}\left(3\right)<10^{-6}$) if we can repair a failed node within 9 h (i.e., $E\left[t_{i}\right]\leq 9$).

## 7. Conclusions

Medical errors in ICUs significantly contribute to patient mortality rates due to the complex health conditions of patients, the urgent need for interventions, and workload variations, making them particularly susceptible to errors. Therefore, there is an urgent need to implement AIoT in the ICU. To illustrate its importance, this paper presents CMUH-RICU as an example of AIoT-enabled data fusion capabilities in hospitals. A four-layer AIoT architecture is proposed to effectively deliver real-time and non-real-time medical data in CMUH-RICU, considering bandwidth, delays, and fault tolerance. The design can process 22 TB of data annually, with an average delay of 1.72 ms and a bandwidth of 65.66 Mbps, sufficient to support all ICUs in the CMUH. In the event of node failures, CMUH-RICU operates normally with a three-node Kafka cluster, ensuring prompt resolution within 9 h for a one-year node lifespan.

A practical example illustrates CMUH-RICU’s ability to fuse real-time medical data into an ARDS AI application, ultimately saving a patient’s life. This showcases the effectiveness of AIoT-driven data fusion in the context of CMUH-RICU. By leveraging AIoT, hospitals can improve patient care and outcomes in ICUs, addressing the critical issue of medical errors. This information fusion strategy significantly improved the medical diagnosis rate, increasing from 52.2% before fusion to 93.3% afterward. Simultaneously, the mortality rate decreased from 56.5% before information fusion to 39.5% afterward.

## Data Availability

The data that support the findings of this study are not publicly available due to privacy, commercialization, and/or ethical restrictions.

## Abbreviations

AI	Artificial Intelligence
AIoT	Artificial Intelligence of Things
APACHE II	Acute Physiology and Chronic Health Evaluation II
ARDS	Acute Respiratory Distress Syndrome
API	Application Programming Interface
AUC	Area Under the Curve
AutoML	Automated Machine Learning
CNN	Convolutional Neural Network
CO	Cardiac Output
DFM	device feature management
ECMO	Extracorporeal Membrane Oxygenation
EKG	Electrocardiogram
FHIR	Fast Healthcare Interoperability Resources
FiO_2_	Inspired Fraction of Oxygen
GUI	Graphical User Interface
GPT	Generative Pre-trained Transformer
HIS	Hospital Information System
HL7	Health Level Seven International
HR	Heart Rate
ICU	Intensive Care Unit
IoT	Internet of Things
JSON	JavaScript Object Notation
LOINC	Logical Observation Identifiers Names and Codes
LTVV	Low Tidal Volume Ventilation
LSTM	Long Short-Term Memory
MLP	Multiple Layer Perceptron
NCLC	no-code low-code
NoSQL	Not only SQL
PACS	Picture Archiving and Communication System
PBW	predicted body weight
PCO_2_	partial pressure of carbon dioxide
PO_2_	partial pressure of oxygen
RESTful	Representational State Transfer
RICU	Respiratory Intensive Care Unit
RR	Respiratory rate
SaO_2_	Arterial Oxyhemoglobin Saturation
SpO_2_	Blood Oxygen Saturation
STEMI	ST Elevation Myocardial Infarction
SV	Stroke Volume
SVM	Support Vector Machine
UCUM	Unified Code for Units of Measure
Ve	Exhalation Minute Volume
Vt	Tidal Volume
XML	Extensible Markup Language
XGB	eXtreme Gradient Boosting
